# Preparation of Nanosized Pharmaceutical Formulations by Dual Centrifugation

**DOI:** 10.3390/ph16111519

**Published:** 2023-10-25

**Authors:** Jonas K. Koehler, Stefanie Schmager, Valentin Bender, Denise Steiner, Ulrich Massing

**Affiliations:** 1 Institute of Pharmaceutical Sciences, University of Freiburg, 79104 Freiburg im Breisgau, Germany; jonas.koehler@pharmazie.uni-freiburg.de (J.K.K.); stefanie.schmager@pharmazie.uni-freiburg.de (S.S.); valentin.bender@pharmazie.uni-freiburg.de (V.B.); 2 Department of Pharmaceutical Technology, Eberhard Karls University Tübingen, Auf der Morgenstelle 8, 72076 Tübingen, Germany; 3 Institute of Pharmaceutical Technology and Biopharmacy, University of Münster, Corrensstraße 48, 48149 Münster, Germany; 4 Andreas Hettich GmbH & Co. KG, 78532 Tuttlingen, Germany

**Keywords:** dual centrifugation, dual asymmetric centrifugation, homogenization, lipid nanoparticles, liposomes, polymersomes, solid lipid nanoparticles, emulsions, nanomilling, pharmaceutical nanotechnology

## Abstract

Dual centrifugation (DC) is an innovative in-vial homogenization and in-vial nanomilling technique that has been in use for the preparation of liposomes for more than one decade. Since then, DC has continuously been developed for preparing various liposomes and other lipid nanoparticles including emulsions and solid lipid nanoparticles (SLNs) as well as polymersomes and nanocrystals. Improvements in equipment technology have been achieved over the past decade, so that DC is now on its way to becoming the *quasi*-standard for the simple, fast, and aseptic production of lipid nanoparticles and nanocrystals in small and medium batch sizes, including the possibility of simple and fast formulation screening or bedside preparations of therapeutic nanoparticles. More than 68 publications in which DC was used to produce nanoparticles have appeared since then, justifying an initial review of the use of DC for pharmaceutical nanotechnology.

## 1. Basic Principles of Dual Centrifugation (DC) and Focus of the Review

DC is a unique process in which a sample vial in a fast-running centrifuge (primary rotation) is additionally turned around a second axis (secondary rotation) [1]. As a result, the direction of the high centrifugal acceleration continuously changes in relation to the (turning) sample vial, which results in highly frequent and, at the same time, strong movements of the sample material inside the vial, which typically contains heavy ZrO-beads to support the process [2]. The very intense sample movements can principally be used for mixing, shaking, milling, or homogenizing. This review focuses on the preparation of lipid and polymer nanoparticles such as liposomes, emulsions, solid lipid nanoparticles, or polymersomes using DC as a tool for in-vial homogenization and of nanocrystals using DC as a tool for in-vial nanomilling.

**Figure 1:**
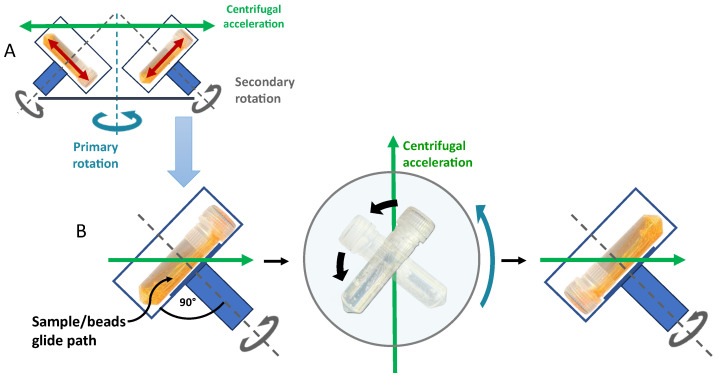
Schematic representation of the basic principles of dual centrifugation using horizontal vial positioning (90° to the second rotation axis). (**A**) Dual rotor with primary and secondary rotation. (**B**) Sample movement in a dual centrifuge due to constant centrifugal acceleration in combination with sample vial rotation. Green arrows show the direction of the constantly effective centrifugal acceleration.

In addition to the high centrifugal acceleration of the samples due to a fast primary rotation and an optimal turning frequency of the sample vial around the second rotational axis, the use of lengthy vials that are placed into the dual rotor at a 90° angle to the axis of the second rotation (horizontal vial positioning, compare Figure 1) is ideal for introducing the maximal energy into the sample material. Due to the very high in-vial homogenization and milling performance, this review is restricted to DC using lengthy vials and the horizontal vial positioning, which, however, includes virtually all previous publications on lipid and polymer nanoparticles prepared with DC.

**Figure 2:**
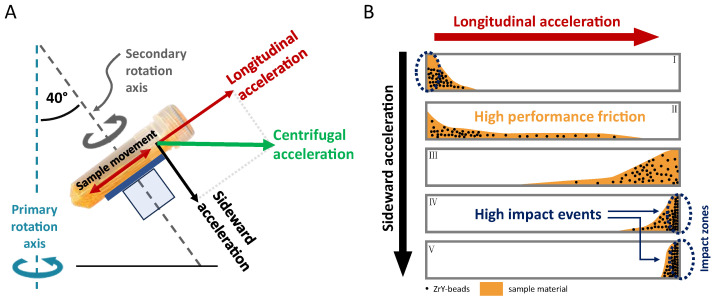
Homogenization principles during DC: (**A**) Centrifugal acceleration during DC is split into a vector that moves the sample along the sample vial (longitudinal acceleration), and a vector that pushes the sample to one side of the inner wall of the sample vial (sideward acceleration). (**B**) Longitudinal acceleration causes sample movement resulting in a strong impact when the mixture of sample and beads reaches the end of the vial (impact zone, right part of the figure, Ⅳ and Ⅴ). In addition to that, the unique interaction of longitudinal and sideward acceleration forces the mixture of sample and beads to a defined glide path while moving from top to bottom of the lengthy vial, which additionally causes high-performance friction (sample glide path, distribution of the sample material is shown in orange color: B, mainly Ⅱ and Ⅲ).

One important aspect explaining the impressive homogenization or milling results is the fact that the horizontal vial orientation gives the sample inside the vial the maximal way to accelerate (Figure 1 and Figure 2), resulting in the strongest impact when the sample containing heavy ZrO-beads reaches the end of the vial. This sample movement is in a certain way comparable to that of a horizontal laboratory shaker, with the important difference that the sample acceleration during DC is more than two orders of magnitude higher and therefore strong enough for efficient homogenization or nanomilling [2]. 

The second important aspect explaining the good homogenization efficacy using lengthy vials in combination with a horizontal vial positioning is that the sample does not “fly” directly from top to bottom. Instead, the sample including the ZrO-beads glides along one side of the inner vial wall from top to bottom and vice versa. Since the axes of the secondary rotation have an approx. 40° angle related to the main (primary) rotational axis (compare Figure 2), the sample gliding takes place on a defined path inside the vials. The explanation for this is that the vector of centrifugal acceleration hits the vessel at this 40° angle and thus acts on the sample in two directions. One vector of the parallelogram of forces pushes the sample from top to bottom of the vial, and the other vector presses the sample on the gliding path (Figure 2). This results in additional “high-performance friction” of the sample by the heavy ZrO-beads.

## 2. Equipment for Dual Centrifugation

### 2.1. Dual Centrifuges

Dual centrifuges that have successfully been used for the preparation of nanoparticles using horizontally orientated vials are the SpeedMixer^®^ DAC 150 (DAC 150, Hauschild GmbH & Co. KG, Hamm, Germany), the ZentriMix 380 R (ZM 380R, Andreas Hettich GmbH & Co. KG, Tuttlingen, Germany), and the DeltaVita 1 (DV1, Erich Netzsch GmbH & Co. Holding KG, Selb, Germany; identical in construction to ZentriMix 380 R).

The SpeedMixer^®^ DAC 150 (DAC 150) was initially designed for very short runs of a few seconds to minutes to mix highly viscous compounds. Thus, this device is equipped with a strong motor that requires a V-belt to couple the main and secondary rotation. Using a V-belt protects the mechanics from slippage when the dual asymmetric centrifuge starts. Despite its maximum run time being 5 min, the DAC 150 can be used to prepare nanoparticles [1] if the necessary run times (typically about 30 min) are reached by multiple short runs. However, standard vial adapters are only available for vertical vial orientation, which is optimal for fast mixing of highly viscous materials such as print inks, silicones, or two-component teeth filling materials [2]. Adapters for the horizontal orientation of small and lengthy vials are not commercially available for the DAC 150 and are all custom made for patent reasons. Since the diameter of the rotational disk to accommodate the sample vial adapter is rather small, only a few vials (typically two) can be processed simultaneously.

**Figure 3:**
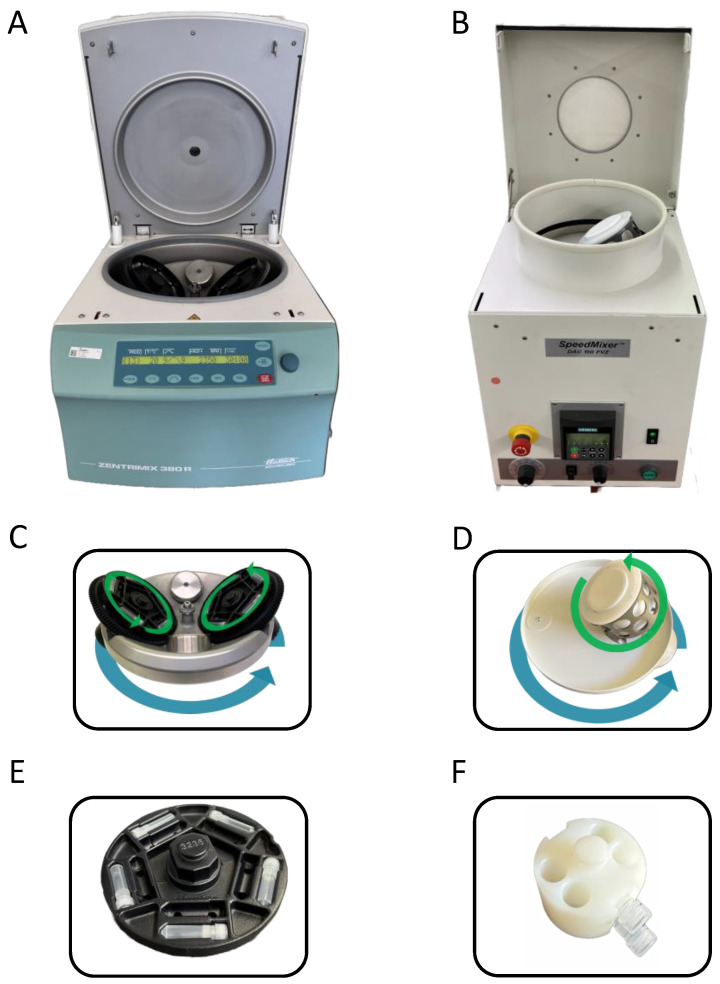
Comparison of DC-devices used for the preparation of nanosized pharmaceutical formulations using the horizontal vial positioning. (**A**) ZentriMix 380 R from Hettich with the corresponding DC-rotor (**C**) and adapter for horizontal positioning of ten 2 mL vials on one level (in total 40 vials in one run possible) (**E**). The ZentriMix-rotor is removeable and can be replaced by a normal centrifugal rotor for using the device as normal centrifuge. The DV1 device from Netzsch is identical in construction. (**B**) DAC 150 from Hauschild with corresponding DAC-rotor (**D**) and customized adapter for horizontal orientation of 2 mL vials (**F**).

A special feature of the DAC 150 is that its rotor is asymmetrically constructed with only one sample holder (Figure 3). Thus, smooth running of this rotor is ensured by a fixed counterweight; as a result, the payload is also fixed to a certain weight. If very small sample quantities are processed, the necessary weight of the payload must be reached by a special (heavy) sample holder. Based on using the DAC 150 with its special asymmetric rotor for the preparation of nanoparticles, in a few publications, the DC-process is named “dual asymmetric centrifugation (DAC)”. In contrast to that, the other dual centrifuges (DV1 and ZM 380R) have symmetric dual rotors with two symmetric sample holders. Therefore, the term “dual centrifugation (DC)” refers to the fact that there are two types of rotational axes within one rotor—one axis for the main rotation of the whole DC-rotor, and a second set of axes for turning the sample vials (compare Figure 3). In the further course of this article, only the term DC is used for the dual centrifugation process as well as the dual centrifuge.

The dual centrifuges DV1 and ZM 380R are designed from the outset to produce nanoparticles, which typically requires longer run times than a few minutes. Thus, the DC-processing time is freely adjustable. Furthermore, the coupling of the main and secondary rotations is established by gear wheels, and the motor control allows a soft start to protect the mechanics. To dissipate the heat inevitably generated during DC-homogenization or -nanomilling, DV1 and ZM 380R are equipped with a powerful cooling unit. Furthermore, dual rotors of DV1 and ZM 380R have rather large rotational disks to accommodate a high number of the typically used 2 mL PP-screw cap vials (maximum 40 vial per run, arranged on 2 levels with 20 vials each), which is advantageous for formulation screening approaches, for example. Due to the frequent change in the direction of the high centrifugal acceleration during DC, all types of vials require special adapters to adequately fix the vials in place (Figure 3E,F).

However, with otherwise equally fast secondary rotation (about 850 rpm at max. speed of main rotation), the centrifugal acceleration of the ZM 380R is about 15% higher compared to the DAC 150, which is due to the larger rotor diameter that allow faster acceleration of the sample materials. Sample materials with higher viscosities thus have a better chance of reaching the end of the vials before the vial has turned again in the opposite direction. Since a significant part of the homogenization or milling processes take place at the end of the sample vials due to collisions (compare Figure 2, impact zones), reaching the end of the vials results in more effective homogenization or milling processes. 

### 2.2. Beads in Use for Dual Centrifugation

Homogenization or nanomilling is typically supported by spherical ceramic beads with a high density (ZrO-beads, above 6.0 kg/dm^3^) since heavy beads accumulate very high kinetic energies during acceleration and thus introduce more energy into the sample material. In the first studies, liposome preparation was performed by DC-homogenization using much lighter glass beads (approx. 2.23 kg/dm^3^ for borosilicate glass), which were used with satisfactory results on first glance [1]. However, it turned out that the numerous collisions between the glass beads resulted in glass wear, which, due to the basic surface properties of the freshly generated glass (nano)particles (containing potassium-oxide), caused degradation of the phosphatidylcholine head groups. The proposed underlying chemical reaction might be the well-known Hoffmann elimination, which started with the removal of a proton in β-position to the quaternary amino group of phosphatidylcholine-species by the basic glass (nano)particles, followed by a rearrangement of bonds. The final reaction product is trimethylamine, which can easily be detected by its fishy smell, even in small quantities [2]. Thus, the use of the much denser and harder ceramic beads was established and almost all studies cited in this review used those beads.

Commonly used ceramic beads are made from zirconium-oxide, which are stabilized with yttrium. An example are SiLibeads type ZY-P Pharma purchased from Sigmund Lindner GmbH (Warmensteinach Germany). Their high degree of roundness (≥0.96 (width to length ratio (x_min_/x_max_)) and polished surface avoids the generation of wear from the commonly used plastic vials (see below Section 2.3). The high hardness (microhardness: ≥1300 HV10) contributes to the avoidance of zirconia wear during bead–bead collisions as well. In a DC-nanomilling study, a zirconium amount of only 3.4 ppm was found in the resulting suspension after 90 min milling time, which was shown to be comparable with wear generated from ZrO-beads during agitator ball milling in pharmaceutical production [3]. 

For DC-homogenization, typical bead diameters range from 0.2 to 1.6 mm. However, the optimal bead size and number (bigger beads) or amounts (smaller beads) vary depending on the specific lipid blend or nanocrystal dispersion and the vial type. Smaller and thus lighter beads introduce less energy during bead–bead collisions but increase the number of bead–bead collisions. Therefore, the bead size and quantity must be optimized for each DC-process.

### 2.3. Vials in Use for Dual Centrifugation

One important aspect of successful dual centrifugation is that the homogenization/milling vials have to be made of a slightly elastic plastic material. In most cases, disposable 2 mL screw cap polypropylene (PP) vials are in use in combination with ZrO-beads. Another important aspect of choosing the right vials for DC concerns their tightness and stability. The vials must be very tightly sealed and stable enough since during DC, the sample material and the beads are pushed with high impact and at high frequency against the lid and the bottom (compare Figure 2). Therefore, it is important to either use the exact vials described in the existing publications or carefully check the tightness of new vials that have not yet been used in test runs. As an alternative, 10 mL PP-injection vials at the same length as the screw cap vials can be used [2].

To our own consideration, wear from the surface of the plastic (PP) vials has not been observed or reported so far when using 2 mL screw cap vials in combination with ZrO-beads of high quality (see above Section 2.2). This could be explained by the high degree of roundness in combination with the smooth, polished surface of the ZrO-beads, which, even when in contact with the plastic walls of the vials, are assumed to not be able to effectively scratch the plastic material. In the case of liposome preparation by DC, the danger of scraping of the plastic walls is further reduced by the lubrication effect of the highly viscous and concentrated lipid blends. Thus far, systematic studies focusing on the generation of wear (PP or ZrO) during the preparation of liposomes within a DC do not exist. 

## 3. Nanosized Pharmaceutical Formulations Made by DC

The following sections focus in detail on the preparation of different nanosized pharmaceutical formulations by DC, namely liposomes, polymersomes, emulsions, solid lipid nanoparticles, and nanocrystals. At the beginning of each section, a brief introduction to the various formulations is given before focusing on their preparation using DC. Depending on the number of references available for the corresponding formulation, they are summarized in a table at the end of each section.

### 3.1. Liposomes and Vesicular Phospholipid Gels (VPGs)

Liposomes are spherical vesicles in which an aqueous core is surrounded by one or more bilayers, which typically consist of amphiphile substances, mostly phospholipids and cholesterol [4,5]. Liposomes in the lower nanometer range are promising and universal drug carriers. Lipophilic drugs and amphiphilic drugs can be embedded in the bilayer structures and hydrophilic drugs inside the aqueous core of the liposomes. An example for the use of drug-loaded liposomes is systemic cancer therapy. After intravenous injection, liposomes tend to accumulate within some cancer tissues due to the enhanced permeability and retention (EPR) effect, thus bringing more of the drug to the cancer and less to healthy tissues, which improves the therapeutic index of the liposomal-entrapped drug [6,7,8].

In many cases, the easiest way to prepare liposomes is the homogenization of an aqueous blend of membrane-forming lipids, which can also contain the (drug)–compound to be entrapped. Classical homogenization tools to produce liposomes are microfluidizers [9], or high-pressure homogenizers (HPH) [10,11] including the French pressure cell [12]. DC represents a completely new type of homogenization technique, which is based on bead–bead-collisions as well as high-performance friction. Since these processes take place in a closed vial, DC-homogenization can also be named as in-vial homogenization. Differences between DC and the other homogenization techniques are found particularly in the minimal and maximal possible batch sizes. Since DC-homogenization requires no dead volume, this technique allows very small batches from the lower mg-range up to medium-sized batches—meaning the lower gram range—to be processed. The other techniques such as HPH require at least gram amounts to be homogenized due to their significant dead volumes and mechanical construction. However, these can process very large batches, especially when used in a continuous mode.

**Figure 4:**
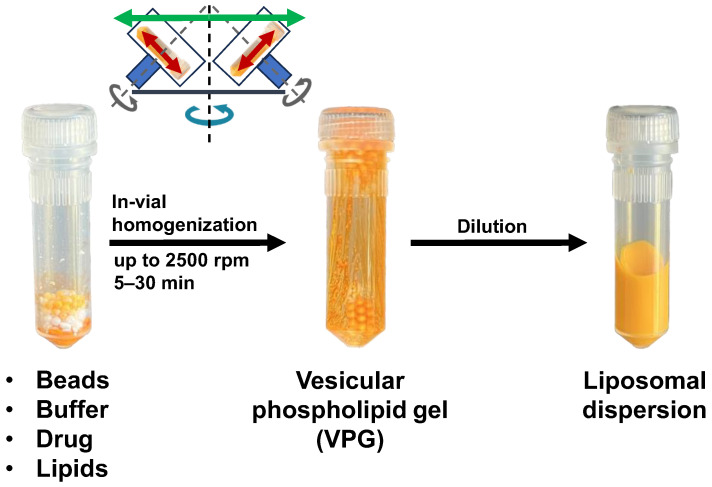
Basic procedure of manufacturing liposomal dispersions by DC.

The general procedure of the DC-preparation of liposomes is shown in Figure 4. A key step is the in-vial homogenization of a rather highly concentrated blend of lipids, typically >10–80% (*w*/*w*), together with an excess of ceramic beads (ZrO). The result is a highly concentrated and thus viscous liposomal dispersion, a so-called vesicular phospholipid gel (VPG) [2,13,14,15]. Within a VPG, the liposomes are very close together with only a minimum of aqueous phase in between [2]. Since the introduction of energy into a lipid blend during processing also depends on its viscosity, high lipid concentrations are of advantage, while lipid concentrations lower than 5–10% (*w*/*w*) cannot be homogenized efficiently using DC [16]. Other homogenization techniques such as HPH or microfluidization allow homogenization at lower lipid concentrations because high shear forces are achieved by forcing the lipid blend through a valve at around 1000 bar or higher. 

After DC-homogenization, the primarily formed VPGs can be diluted to a conventional liposomal formulation by simply adding buffer, followed by mechanical agitation, which can easily be performed with the same DC at low speed [1]. Simple vortexing is also possible. Alternatively, VPGs can be used as they are, e.g., as a liposomal storage form (dilution at a later time point) or as depot formulation for direct application [15]. 

However, despite the peak energy that can be introduced into a lipid blend by DC being clearly lower compared to HPH or microfluidization, DC-homogenization of the same blend of lipids results in highly comparable liposomes. An explanation is that DC-homogenization is a continuous process, consisting of numerous consecutive homogenization events. At maximum speed, about 50,000 stressing events take place within a typical 30 min DC-run without breaks in between [1,16,17,18,19]. In contrast, HPH-homogenization typically consists of 10 separate homogenization steps (cycles) [20]. The peak energy during each HPH-step is very high, but only affects the sample for a very short time, in the millisecond range. However, due to the much lower peak energy in combination with a much higher number of consecutive homogenization events, the stressing in the DC is less intense compared to HPH or microfluidization and therefore ideal to entrap sensitive drug compounds such as nucleic acids [21,22]. The entrapment of sensitive drug compounds is also facilitated by the fact that cooled DCs have been available for some years now (see above—available equipment). Furthermore, hydrolysis of phospholipids is greatly reduced during DC-homogenization [1].

An interesting feature of DC-homogenization over long-established liposome preparation methods such as membrane filter extrusion [23,24], sonication [4], or ethanol injection [25] is the possibility of completely avoiding organic solvents to prepare a molecular dispersed lipid mixture prior to homogenization [16,18]. Thus far, and especially when using the non-water-soluble cholesterol as part of the liposomal lipid composition, a molecular dispersed lipid mixture (a so-called lipid film) must be prepared prior to liposome preparation. For that, the different lipids must be dissolved together in organic solvents, followed by a solvent removal step. In contrast, when liposomes are prepared with DC, a simple blend of the dry lipids can be used, and the homogeneous distribution of the cholesterol within the phospholipid membranes takes place during the DC-process [16,18]. The explanation is that during DC-homogenization, DC-nanomilling of the cholesterol crystals also occurs, which increases the solubility of cholesterol [16]. Once dissolved, cholesterol molecules can easily be embedded within the phospholipid bilayer. This is an important advantage of the DC-preparation of liposomes, which saves time and resources, and avoids the possibility of organic solvent residues in the liposomal formulation. Khadke et al. also described a solvent-free preparation of liposomes using a high-shear mixing device. However, the resulting liposomes had to be further size reduced by a following HPH-step (two-step method). Due to the rather high volumes necessary for using the high-shear mixing device, the preparation of small batches of liposomes is not possible using this method [26]. 

#### 3.1.1. Morphology of DC-Made Liposomes

**Figure 5:**
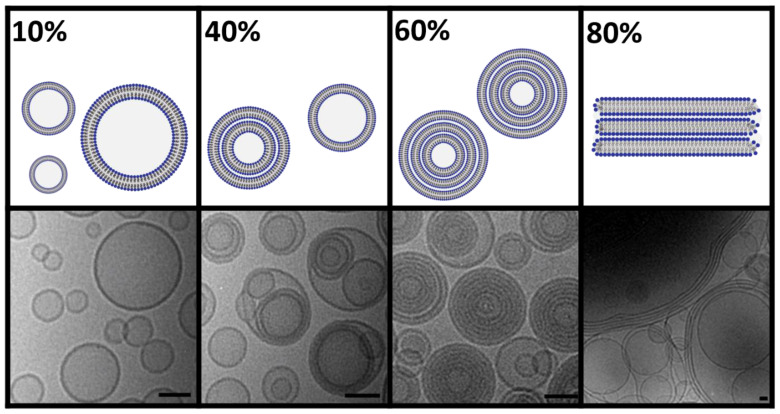
Morphology of HEPC/Chol 55/45 mol% liposomes prepared by DC in dependency of the lipid concentration used for DC-homogenization. Schematic illustration of unilamellar (10%), uni- and multilamellar liposomes (40%), multilamellar (60%), and open lamellar lipid stacks (80%). Scale bar 50 nm. Cryo-EM images adopted from Koehler et al., Pharmaceutics, 2023 [18].

In contrast to the long-established liposome preparation methods such as extrusion, sonication, or ethanol injection, DC allows controlled tailoring of the lamellarity of the liposomes by adjusting the lipid concentration during homogenization (Figure 5) [18]. At low lipid concentrations (approx. 10% *w*/*v*), the viscosity of the lipid/water mixture is rather low, resulting in a weaker introduction of energy during DC and thus, slightly larger and more heterogenous, unilamellar liposomes. Above a lipid concentration of 20% (*w*/*v*), DC-homogenization becomes more effective and results in rather small unilamellar liposomes with a further decrease in vesicle heterogenicity by increasing lipid concentrations (lower PDI-values) [18]. 

At lipid concentrations of >30%, more lipids than necessary are available for forming the maximum tight package of small unilamellar vesicles. Thus, excess lipids go into the vesicles, forming small multilamellar vesicles (SMV) [18]. While lamellarity continues to increase with increasing lipid concentration, the vesicle sizes remain small, but the vesicles become more uniform. This reduction in size distribution with increasing lipid concentration during DC-homogenization is probably the result of the higher mechanical stability of the resulting SMVs against shear stress. At about 30% lipid concentration, the entrapping efficiency (EE) for water-soluble compounds reaches its maximum of typically >50%, which remains constant at further increasing concentrations. Due to its high water binding capacity, the addition of PEG-lipids to the liposomes leads to an overall lower lamellarity and EE [16,18].

At very high lipid concentrations, there is not enough water available to fully hydrate the phospholipid headgroups. Thus, a further increase in the lipid concentration (>60%) results in an increasing ratio of open lipid stacks within the primarily formed VPGs [16,18].

#### 3.1.2. Current DC-Applications in Liposome Research

The preparation of liposomes is currently the most frequent application using DC reported in the literature, summarized in Table 1. The table is divided into categories according to the active ingredient used (antibiotics, cytostatics, peptides, RNAs, model drugs) and studies, where the focus was on physical investigations of liposomes.

Reviewing the literature, it was shown that calcein is a very common hydrophilic model drug substance that can be entrapped within the hydrophilic core of liposomes. Its high passive entrapment into DC-made liposomes (up to 56% EE) was first shown in 2007 [1]. However, similar or even higher EE-values could also be found for real drug compounds such as the water-soluble cytostatic cytarabine (up to 56%) [27] or for a siRNA (up to 71%) [21]. Additionally, rather lipophilic drugs such as chloramphenicol could be entrapped with comparable high EE-values of up to approx. 56% as shown by Ingebrigtsen et al. [28]. Another antibiotic that could be entrapped in liposomes using DC is the antibiotic peptide vancomycin. Due to the entrapment into liposomes containing tetraether-lipids, the oral bioavailability of the peptide was significantly improved [29,30].

The always very high passive drug entrapment of lipo- and hydrophilic drug compounds processed with a DC allows in certain cases a potential clinical use of DC-made liposomes without the purification and removal of the non-entrapped drug compound [16]. Usually, non-entrapped drugs are removed from the liposomal formulation by a purification step (such as size exclusion chromatography, centrifugal concentrators, ion exchanger, tangential flow filtration), which is logical, especially when only a minor part of the drug could be entrapped [31,32,33,34]. The high passive entrapment during DC-processing of up to approx. 70% also makes the use of the non-purified liposomal dispersion possible, especially if the free drug is either rapidly degraded in the systemic circulation after application (e.g., RNA, gemcitabine) or if the non-trapped drug is non-toxic or not active outside its target (e.g., RNA) [20].

Effective cancer treatment is often limited by the insufficient delivery of anticancer drugs due to their poor solubility or due to a non-effective entrapment into nanocarriers. In the case of docetaxel, DC-processing turned out to be a promising approach to entrap the poorly soluble drug with high entrapping efficiency into liposomal vesicles [35].

Cytostatic drugs often imply issues of undesirable side effects due to their high cytotoxicity and no specificity. To overcome these side effects, the processing of the formulation in DC implies the possibility of using undiluted VPGs as a local depot formulation to gain a sustained release for anticancer drugs as shown by Qi. et al. [36].

In addition to the possibility of efficiently entrapping small drug molecules, the rather gentle DC-process also allows the entrapment of bigger and fragile compounds such as protein-based molecules or nucleic acids. This has been demonstrated for rather large protein-based molecules such as EPO [13,15], as well as for shorter peptides such as exenatide [37]. Entrapping of these molecules into a VPG by using a DC resulted in a controlled and sustained release of the macromolecules without affecting their integrity [37].

Since the COVID-19 pandemic, RNA therapeutics have been of extraordinary interest for the treatment of this new viral infection (vaccine), but also for use in existing diseases, especially to support cancer immune therapy. Even before the pandemic, DC proved to be a suitable technique to formulate small non-coding RNA such as siRNA [21,22,38]. Furthermore, formulation of the larger and more fragile mRNA into LNP-like vesicles could also be achieved [39].

**Table 1:** Liposomes prepared by DC.

Category	Drug	Lipid Composition	DC Device	Smallest Measured Size	Highlight	Reference
Analgesic	Mifepristone, FKBP51 ligand SAFit2	E80	DAC 150	not applicable	SAFit2 encapsulated in a DC-made vesicular phospholipid gel (VPG) can be used to target the stress regulator FKBP51.	[40]
Antibiotics	Chloramphenicol	S100	DAC 150	approx. 130 nm	Liposome-in-hydrogels were made by DC using soy phosphatidylcholine and water-soluble β-1,3/1,6-glucan as gel component for the delivery of chloramphenicol.	[41]
	Benzoyl peroxide and chloramphenicol	S100	DAC 150	132 nm	DC-based co-encapsulation of benzoyl peroxide and chloramphenicol into liposomes without detectable influence of the two drugs on liposomal characteristics or release profiles.	[42]
	Chloramphenicol	E80/Phospholipon^®^ 90H/S100/Soluthin^®^ S90	ZM 380R	120 nm	DC-preparation of a liposome-in-hydrogel using various lipids and chitosan. High entrapment of chloramphenicol.	[28]
	Vancomycin	Lecithin/Chol/GCTE	DAC 150	95 nm	DC-preparation of a promising drug delivery system for oral application of peptide drugs. Potency of the drug was proven using a rat model. Long-term stable formulation was achieved.	[29]
	Vancomycin	EPC/Chol/head-group-modified PL	DAC 150	87 nm	Promising approach to overcome the mucosal barrier. Using DC-prepared liposomal nanocarriers carrying cell-penetrating peptides for oral delivery of vancomycin.	[30]
Antiviral drugs	Myrcludex B	GCTE	DAC 150	approx. 130 nm	GCTE-liposomes for efficient oral administration of Myrcludex B. Long-term storage possible due to freeze-drying.	[43]
Chelating agent	Copper-chelating Trientine	EPC/Chol/DSPE-mPEG_2000_/DSPE-PEG_2000_ Maleimide	DAC 150	139 nm	Triethylenetetramine (TETA) in DC-prepared vectorized liposomes showed an up to 16-fold higher brain uptake in in vivo experiments in rats compared to free TETA or TETA in non-vectorized liposomes.	[44]
Contrast agents	Superparamagnetic iron oxide particles	DPPC/POPC/DPPE m-PEG_2000_/Chol/DSPE-PEG_2000_ Maleimide	ZM 380R and DAC 150	127 nm	Immuno-magnetoliposomes (ML) with a high amount of entrapped superparamagnetic iron oxide particles (SPIOs) were prepared by DC. Immuno-ML can target activated platelets and are thus potentially suitable as MRI contrast agent for the detection of ruptured plaques.	[19]
Cytostatics	Docetaxel	S100/Chol/DC-Chol/DMPE/DMPG/DOPE/DMPC/DPPG/DOPC/DOTAP/POPC/POPE/DSPE-PEG_2000_/DSPE-PEG_750_	DAC 150	58 nm	Various lipid compositions were tested to identify liposomal lipid compositions for effective docetaxel entrapment.	[35]
	Mitotane	DOPC	ZM 380R	117 nm	Final MT concentration 0.67 ± 0.01 mg/mL; stable for 6 months at 4–8 °C.	[17]
	Cytarabine	E80	DAC 150	163 nm	Viscosities of VPGs were enhanced by coating the liposomes with cationic or anionic polyelectrolytes. Very slow in vitro release of cytarabine from the VPGs (up to 18 days).	[36]
	Mitotane, Everolimus	DOPC/POPC/DSPC/Chol	ZM 380R	130 nm	Simultaneous analysis of hydrophobic drugs and lipids in DC-made liposomes by HPLC-DAD-CAD.	[45]
Liposomal studies	Calcein	EPC/Chol/DSPE-PEG_2000_	Rotanta 400 DC prototype	110 nm	Cholesterol–polymer amphiphiles were used for steric stabilization of DC-made liposomes. Click chemistry was used for conjugation of small molecules to the liposomal surface.	[46]
		EPC/Chol/DSPE-PEG_2000_	Rotanta 400 DC prototype	130 nm	Functionalization of DC-made liposomes by click chemistry.	[47]
	Indocyanine green	EPC/DPPG/Chol/DSPE-PEG_36_	DAC 150	216 nm	Matrix-assisted laser desorption/ionization mass spectrometry is used for ex vivo imaging of liposomal carriers in mouse tissue. Indocyanine green serves as cargo and DPPG/PEG36-DSPE as lipid marker.	[48]
		EPC3/DPPC/DSPC/20:0 PC/Chol	ZM 380R	162 nm	Migration of DC-made liposomes modified with fluorescence-labeled conjugates of different lengths into biomembranes.	[49]
		HEPC/Chol/DSPE-PEG_2000_/DPPC	ZM 380R	approx. 100 nm	Small multilamellar vesicles (SMV) can be prepared by DC in a highly reproducible way.	[18]
	Calcein	HEPC/Chol	DAC 150	approx. 50 nm	It was shown for the first time that DC can successfully be used for the preparation of VPGs and liposomes.	[1]
	Vancomycin, Insulin	Lecithin/Chol	DAC 150	124 nm	It has been demonstrated that potential cross-reactions between macromolecular drugs and activated lipids during DC-preparation of liposomes cannot be neglected.	[50]
	Membrane dye DiI	EPC/Chol/DSPE-mPEG	Rotanta 400 centrifuge with a prototype DC-rotor	approx. 100 nm	Investigation of the protein corona of DC-prepared liposomes (unfunctionalized vs. PEGylated vs. hyperbranched polyglycerol functionalized) and their influence on uptake by macrophages.	[51]
	Calcein-AM, Tamoxifen	Essential phospholipids/polyenylphosphatidylcholine (PPC)/phosphatidylinositol (PI)	ZM 380R	not specified	The effect of essential phospholipids (EPL) on hepatocyte function in vitro was investigated and valuable insights into the mechanism of action of EPL were gained.	[52]
		EPC/DSPE-mPEG_2000_/DSPE-PEG_2000_ Maleimide/DPPE-RH/Chol	DAC 150	118 nm	DC-made liposomes are conjugated with cationized bovine serum albumin as a transport vector to penetrate the blood–brain barrier (BBB).	[53]
Lyso-PC	Saturated and mono-unsaturated Lyso-PC	DSPC/Chol	DAC 150	not specified	Liposomes made by DC using saturated phospholipids caused an increase in saturated lyso-phosphatidylcholine (Lyso-PC) in plasma of tumor bearing mice, which caused a decrease in metastases.	[54]
Model drug	Carboxyfluorescein	E80/Chol	ZM 380R	134 nm	An ex vivo rat intestine model is used to investigate the effects of DC-made matrix-liposomes on intestinal tissue.	[55]
	Calcein	HEPC/EPC/Chol/DSPE-PEG_2000_	ZM 380R	approx. 100	Liposomes can be prepared by DC without lipid film preparation. Encapsulation efficiency can be determined without separation of the free calcein.	[16]
Peptide/Protein-based	Fluorescein isothiocyanate-dextran (FITC), 5(6)-carboxyfluorescein (CF)	E80/SPC	DAC 150	approx. 200 nm	DC-made matrix-liposomes for controlled peroral delivery of peptides.	[56]
	Human growth hormone (hGH), Omeprazol	EPC/Chol/RH-DPPE	DAC 150	203 nm	Oligolamellar vesicles for oral delivery of human growth factor were prepared by DC.	[57]
	Erythropoietin (EPO)	E 80/DOTAP/DPPA	DAC 150	approx. 160 nm	VPGs are presented as promising alternative depot systems for protein drugs.	[15]
	EPO	E80	DAC 150	not applicable	Needle-free injections of DC-made VPGs with various phospholipid contents were tested. Pig skin as an in vitro model as well as gelatine blocks were used for the release studies.	[13]
	Granulocyte colony-stimulating factor (G-CSF)	E80	DAC 150	not applicable	DC-made VPGs were used for controlled release of G-CSF. A continuous release over > 4 weeks could be observed in vitro.	[58]
	Exenatide	POPC/POPG	DAC 150	not applicable	Investigation of DC-prepared VPGs made from POPC and/or POPG carrying exenatide. The release of exenatide, as well as the release of phospholipids were investigated in vitro.	[37]
	Vancomycin, Exenatide	Lecithin/Cholesterol/Glycerylcaldityl tetraetherlipid/cell-penetrating peptide-phospholipid conjugate	DAC 150	122 nm	DC-prepared liposomes containing a cell-penetrating peptide achieved high oral bioavailability for the peptides vancomycin and exenatide.	[59]
RNA	siRNA	HSPC/POPC/DDAB/DSPE-PEG_2000_	DAC 150	185 nm	DC-prepared liposomes containing siRNA were modified by sterol-based post-insertion technique (SPIT) to couple anti-GD2 antibodies for targeting neuroblastoma cells.	[22]
	Calcein or siRNA	EPC-3/S80/DMPG/DPPG/Chol/DSPE-mPEG_2000_	DAC 150	79 nm	Investigation of siRNA integrity by FRET during liposomal preparation by DC.	[21]
	siRNA	EPC-3/Chol/DSPE-mPEG_2000_	DAC 150	approx. 100 nm	FRET-based visualization of fluorescence-labeled siRNAs in cells after microinjection, lipoplex-mediated transfection, and liposome-mediated transfection.	[38]
	Luciferase-coding mRNA	DOTAP	DAC 150	147 nm	DC-prepared hybrid nanoparticles from lipidic and polymeric components were used for the mRNA-transfection. Different ratios of the cationic lipid DOTAP and the cationic biopolymer protamine were compared.	[39]

**Abbreviations**: **PC**—phosphatidylcholine; **E80**—egg PC 80%; **S100**—soy PC 100%; **Chol**—cholesterol; Phospholipon^®^ **90H**—hydrogenated PC ≥ 90%; Soluthin^®^ **S90**—soy lecithin, min. 83% associated with calcium chloride; **GCTE**—Glycerylcaldityl tetraether; **EPC**—egg PC; **PL**—phospholipids; **DSPE-PEG_2000_**—1,2-distearoyl-sn-glycero-3-phosphoethanolamine-N-[amino(polyethylene glycol)-2000]; **DSPE-mPEG_2000_**—distearylphosphatidylethanolamine-methoxypolyethylene glycol-2000; **DSPE-PEG_2000_ Maleimide**—Distearylphosphatidylethanolamine-polyethylene glycol-2000 maleimide; **DPPC**—1,2-dipalmitoyl-sn-glycero-3-phosphocholine; **POPC**—1-palmitoyl-2-oleoyl-glycero-3-phosphocholine; **DPPE m-PEG_2000_**—1,2-Dipalmitoyl-sn-glycero-3-phosphoethanolamine-N-[methoxy(polyethylene glycol)-2000; **DC-Chol**—3ß-[N-(N’,N’-dimethylaminoethane)-carbamoyl]cholesterol; **DMPE**—1,2-dimyristoyl-sn-glycero-3-phosphoethanolamine; **DMPC**—1,2-dimyristoyl-sn-glycero-3-phosphocholine; **DPPG**—1,2-dipalmitoyl-sn-glycero-3-phosphoglycerol; **DOPC**—1,2-dioleoyl-sn-glycero-3-phosphocholine; **DOTAP**—1,2-dioleoyl-3-trimethylammonium-propane; **POPC**—1-palmitoyl-2-oleoyl-sn-glycero-3-phosphocholine; **POPE**—1-palmitoyl-2-oleoyl-sn-glycero-3-phosphoethanolamine; **DSPE-PEG_750_**—1,2-dioleoyl-sn-glycero-3-phosphoethanolamine-N-[methoxy(polyethylene glycol)-750]; **DSPC**—1,2-distearoyl-sn-glycero-3-phosphocholine; **DSPE-PEG_36_**—1,2-distearoyl-sn-glycero-3-phosphoethanolamine; 20:0 **PC**—1,2-diarachidoyl-sn-glycero-3-phosphocholine; **HEPC**—hydrogenated egg phosphatidylcholine (trade name Lipoid GmbH **EPC3**); **RH**—rhodamine; **DPPE**—1,2-dipalmitoyl-sn-glycero-3-phosphoethanolamine; **DPPA**—1,2-dipalmitoyl-sn-glycero-3-phosphate; **POPG**—1-palmitoyl-2-oleoyl-sn-glycero-3-phosphatidylglycerol; **DiI**—1,1′-dioctadecyl-3,3,3′,3′-tetramethylindocarbocyanine perchlorate.

#### 3.1.3. Discussion—DC-Preparation of Liposomes/VPGs and Their Applications

The growing use of a DC in liposome preparation can first be explained by the exceptional simplicity and speed of the process. Since the homogenization process takes place in a tightly closed vial, DC-homogenization is intrinsically safe, and thus suitable to also entrap toxic compounds safely such as cytostatics. Starting the homogenization with sterile vials and lipids in a DC, an aseptic liposome preparation also needs no further efforts. However, since DC is an in-vial process, continuous preparation or upscaling is not possible.

Due to the rather high lipid concentrations used during DC-homogenization, EE-values for water-soluble compounds are unusually high, so in some cases, the removal of the non-entrapped cargo appears to be not necessary. Adjusting the lipid concentration during DC-preparation of liposomes allows either unilamellar or small multilamellar vesicles (SMVs) to be prepared independently, with the latter having a very low size distribution [18]. SMVs are expected to be promising carriers for lipophilic drug compounds [60].

Thus far, the minimum batch size for DC-made liposomes is approx. 1 mg, which also makes DC-homogenization attractive for the preparation of liposomes with expensive or rare compounds, e.g., biological experiments including studies with small animals [2,47]. Furthermore, a “just in time preparation” of sterile, drug-carrying liposomes for clinical use (bedside preparation) is also possible, which relativizes the problem that no continuous liposome preparation is possible using DC. Such a strategy would overcome problems with the sometimes not sufficient liposomal shelf-lives. Bedside preparation of liposomes for clinical use can easily be performed in a clinical pharmacy by simply adding a defined volume of water/buffer to a sterile and dry blend of lipids and drug in a suitable vial, followed by DC-homogenization, dilution, and dosing in an infusion bag. Such a strategy is even more attractive since the preparation of a lipid film prior to DC-homogenization is not necessary, which saves time and organic solvents.

As an alternative to bedside preparations of liposomes, drug loaded VPGs, which can either be prepared by HPH or by DC, can be used as an improved storage form. These formulations can easily be diluted to a normal liposomal dispersion shortly prior to use. Since there is no concentration gradient between the drug in the aqueous core and outside the liposomes within a VPG, EE-values remain stable even for drugs that can (slowly) diffuse through liposomal membranes [15]. Further investigations are needed to improve the new concepts of preformulated VPGs or of bedside preparation of VPGs/liposomes.

### 3.2. Polymersomes

Polymersomes are the polymer-based counterparts of the lipid-based liposomes and consist of nanoscale block copolymer vesicles with an aqueous core. They can be used as drug carrier systems for both hydrophilic and hydrophobic cargo [61]. Various studies have shown that DC is also suitable for the preparation of polymersomes from different types of copolymers. Köthe et al. described the preparation of polymersomes with the widely studied diblock copolymer PEG-b-PCL (poly(ethylene-glycol)-block-poly(ε-caprolactone)) [62]. Their preparation method is based on the DC-method developed for liposomes. After a polymer film is prepared from the polymer solution by removing the remaining organic solvent under vacuum, DC is used in combination with ceramic beads to homogenize the polymer film with the added PBS (phosphate-buffered saline solution). Several DC-experiments were performed to determine the optimal bead mass in the vials and the processing time. With this method, particle sizes of 147 nm could be achieved, and an entrapping efficiency of a hydrophilic drug of approx. 35% was obtained. Depending on the drug substance, the vesicles were loaded by adding the drug before the polymer film was produced or before the DC-homogenization step.

DC was also used for the preparation of polymersomes from amphiphilic P(DHPMA)-based block copolymers [61,63], polypeptide-block-polypeptoid copolymers [64], pGlu(OBn)-block-pSar [65,66], and copolypept(o)ides [67]. These formulations had in common that the polymers must be incubated in purified water, PBS, or a PBS-based solution of the cargo to be encapsulated and then processed by DC using ceramic beads to intensify the stress conditions. While Scherer et al. [61] developed a method with which the samples were homogenized for 2 × 16 min and then for a further 2 × 2 min after the addition of additional PBS, the homogenization time was reduced to once period of 20 min by the following studies [67]. These procedures resulted in very small vehicle sizes of approx. 37 to 98 nm. The drug molecules not entrapped in the polymersomes were subsequently removed by, e.g., size exclusion chromatography [61,64] or spin filtration [67]. Scherer et al. reported entrapping efficiencies of up to 40% using DC, which was significantly higher than using a conventional method for drug loading [61]. Fenaroli et al. used the polymersomes to study their accumulation in tuberculosis granulomas in zebrafish embryos and mice. They found that the nanoparticles accumulate more in sections of the lung infected with tuberculosis than in uninfected parts [65], which makes them interesting as a drug carrier for the treatment of tuberculosis. To improve the bioavailability and success of tuberculosis treatment, Dal et al. entrapped four second-generation pretomanid derivatives into polymersomes made of pGlu(OBn)27-b-pSar182 block copolymer. The resulting so-called π-π-PeptoMicelles are stabilized by π-π interactions between aromatic groups in the hydrophobic polypeptide block and with the electron-deficient aromatic systems in the applied drugs, and are even stable in the presence of blood plasma and lung surfactant [66]. 

The above-mentioned studies show that DC is well suitable for the preparation of polymersomes. Vesicles could be made from polymers that could not be prepared by other methods, and high polymer concentrations could be processed resulting in high entrapping efficiencies [61,62,63,64].

### 3.3. (Lipid) Emulsions

In pharmaceutical formulations, (lipid) emulsions have long been of interest, and not only for parenteral nutrition. Lipid nanoemulsions in particular are suitable as delivery systems for lipophilic drugs. While high-pressure homogenization is the most common manufacturing method for (nano)emulsions, DC proved to be a suitable alternative, especially when small batch sizes are preferred [68,69,70].

With the aim of preparing parenteral soybean oil emulsions without loss of substance in a short time, Tenambergen et al. were the first to use DC for emulsification. Emulsions with a sample volume of 5 mL were prepared in three separate DC-steps without the aid of beads. It was found that high viscosities of the formulation were favorable in this setup, so the first two DC-steps were performed with only 4% and 8% of the aqueous phase, respectively, while the remaining aqueous phase was added before the last step. This bead-free DC-emulsification achieved droplet sizes of 0.87 µm, which were found to be stable over a period of 6 months [69]. Later, DC-emulsification was further developed into a single-step process using DC without beads. For topical application, a poloxamer-based gel with a triacetin oil phase as a vehicle for the drug pirfenidone was prepared within 2 min with droplet sizes of approx. 250 nm [70]. Droplet sizes of approx. 200 nm were achieved when a curcumin-containing MCT emulsion (medium-chain triglycerides) was emulsified in DC using ZrO-beads to improve the shear stresses during processing [68]. 

Previously, not only lipids in an aqueous phase were emulsified using a DC, but also molten cannabidiol [71] or perfluorocarbon [72] to prepare sterile nanoscale formulations on a small scale. In both studies, ZrO-beads were used to introduce high shear stresses into the formulations, resulting in droplet sizes below 200 nm.

### 3.4. Solid Lipid Nanoparticles

In addition to lipid nanoemulsions, lipid nanosuspensions (solid lipid nanoparticles) were used in formulation development to improve the bioavailability of poorly water-soluble drugs. The suspensions are mainly prepared by emulsification of the molten lipid and droplet crystallization afterwards. To date, high-pressure homogenization has been the preferred manufacturing process for solid lipid nanoparticles. However, this method requires a two-step sample preparation: separate heating of the aqueous as well as the lipid phase above the melting point of the lipid followed by a pre-emulsification step of both phases prior to HPH, which must also be performed at temperatures above the melting point of the lipid to prevent particle crystallization. Recent studies have shown that these manufacturing steps can be combined to only one by using a DC, which greatly simplifies and accelerates the preparation of solid lipid nanoparticles [73]. In order to achieve sample temperatures above the melting point of the lipid, a prototype DC based on the ZentriMix 380 R was equipped with a heating coil. This allowed the process chamber to be preheated up to 60 °C.

In various studies, the DC-based emulsification of three different triglycerides, trimyristin, tripalmitin, and tristearin (melting temperatures between 56 °C and 73 °C) were investigated in 2 mL screw cap vials. Ceramic beads were used to provide sufficient mechanical stress to support the emulsification. It was shown that preheating of the DC and the following emulsification resulted in sample temperatures above 75 °C within 5 min, which showed that an additional sample heating before DC-emulsification is not necessary [73]. With regard to the further course of temperature in the vials over time, it was found that the DC-emulsification had to be stopped after 10 min, as the sample temperatures were then above 90 °C. This could cause the disposable 2 mL screw cap vials to burst due to the increasing pressure in the samples. However, measurements of the particle sizes of the cooled lipid nanosuspensions showed that sizes below 200 nm and PDI-values (polydispersity index) of less than 0.2 could be obtained, indicating monomodally distributed lipid nanosuspensions [73,74]. While the evaluation of various process parameters such as the size and amount of the ceramic beads indicated a rather low influence on the resulting particle fineness (process time 10 min), high process temperatures proved to be advantageous, especially with regard to the width of the particle size distribution. A more pronounced effect on the particle size distributions was observed when the viscosity of the lipid suspension was increased. This was obtained by increasing the lipid and emulsifier content in the formulations. For a trimyristin dispersion, a particle size of 110 nm and a PDI of 0.09 was achieved using this strategy [73].

In another study, the DC was used for stabilizer screening experiments to prepare lipid nanosuspensions. Different types of triglycerides as well as additives in combination with and without surfactant were DC-emulsified for 10 min using ceramic beads and a preheated DC. This proved that DC is a very time- and material-saving screening method, and the results showed that a scaling up to HPH using the same combination of triglyceride and additives was possible [75].

### 3.5. Nanocrystals

DC using horizontally placed vials can also be used for wet ball (bead) nanomilling of poorly soluble drugs to nanocrystals. Nanomilling is applied to increase the solubility of the drugs and thus their bioavailability, which is a key factor for the success of a pharmacotherapy. To ensure the stability of the resulting nanocrystals, stabilizers such as surfactants and polymers must be added prior to nanomilling to prevent particle agglomeration and Ostwald ripening [76,77].

Before a DC was introduced for wet ball nanomilling in 2017, nanomilling studies were often performed using classical planetary ball mills such as the Retsch PM100, Pulverisette 7 from Fritsch or the Nanopulverizer NP-100 from Thinky, to name just a few. DC offers several advantages over using these classical planetary ball mills for nanomilling. Classical planetary ball mills primarily differ from the DC-approaches reviewed here in terms of the size of the milling containers, which are typically made of the same material as the milling balls and their orientation. Because the containers are vertically oriented, efficient wet ball nanomilling with planetary ball mills usually only works with bigger containers and thus larger sample sizes. However, a modified planetary ball mill with small containers of 0.05–1.0 mL volume has been developed, which allows formulation screenings with small sample volumes, but this method needs significant more time than the comparable DC-process [78,79,80]. Using DC and horizontally oriented vials instead allows batch sizes down to 10 mg, which is a huge advancement when only small amounts of a new compound are available and nanomilling conditions must be investigated from scratch. Furthermore, very small and sterile batches of nanocrystals can be prepared with DC for early biological studies, including animal experiments.

As well as the above-described advantages of DC-nanomilling such as the very small batch sizes and the high number of parallel samples, the question arises as to whether DC is as effective as classical wet ball milling [81]. To investigate this question, Hagedorn et al. compared the particle sizes and size distributions obtained from both DC-milling as well as planetary wet ball milling. Nanomilling of low active pharmaceutical ingredient (API) concentrations of 10% naproxen resulted in similar particle sizes and size distributions for both DC- and planetary wet ball nanomilling, while the DC was clearly more effective in milling ibuprofen and fenofibrate suspensions. Here, DC-nanomilling led to significantly smaller particles and narrower size distributions. Important for the protocol transfer to bigger industrial mills (agitator mills) was the finding that DC-nanomilling is suitable to produce consistent small particles and size distributions even with higher drug concentrations of up to 40% of naproxen, while planetary wet ball milling resulted in significantly larger particles at concentrations higher than 10% API, highlighting the limitations of the planetary wet ball nanomilling approaches at higher viscosities. Hagedorn et al. concluded that the introduction of energy is higher for DC-nanomilling since energy is not only introduced into the sample when the cloud of beads is abruptly stopped at the end of the vial with the sample material in between but also when the cloud of beads homogenize the sample while gliding along the vial wall in a friction-like manner [81] (compare Figure 2). Most probably due to that reduced energy intake, the run times of planetary ball mills for successful nanomilling are rather long, e.g., in total 7 h (14 cycles of 30 min each interrupted by cooling breaks of 5 min) in comparison to 1.5 h for DC in the above-mentioned study.

Nanomilling using DC was first introduced in 2017 by Hagedorn et al. for a fast screening of optimal nanomilling conditions, such as the concentration of the active ingredient, and the type and amount of excipients (surfactants, polymers), as well as the amount and diameter of ZrO-beads [81]. It was shown that nanomilling is easily possible using small and lengthy PP-vials and rather small ceramic beads, even with very low quantities of poorly water-soluble drugs of about 10 mg. In this study, up to 40 samples were nanomilled in parallel within 90 min and a DoE-approach was used to define the optimal ratio of drugs and excipients.

In a follow-up study, it was shown that optimal milling conditions screened by using a DC- and DoE-approach could be transferred to huge industrial agitator mills resulting in virtually identical nanocrystals. That the same milling limit could be achieved for both DC- and agitator nanomilling showed that the milling energy of DC is comparable to that of an agitator mill [3].

Due to the above-mentioned advantages for industrial process development, DC-nanomilling has become especially attractive for the pharmaceutical industry. For example, Willmann et al. demonstrated that DC-nanomilling is suitable for facilitating early formulation development of itraconazole nanosuspensions [82]. In another study, DC was used to develop a nanosuspension of centella asiatica extract to optimize the drug delivery properties and its side-effect profile [83]. Recently, Zulbeari et al. showed that nanomilling using DC leads to highly reproducible particle size distribution and stability data for screening approaches. Furthermore, DC was used to develop subcutaneous injectable rotigotine nanosuspensions in aqueous and also in oily media to prolong the drug release. For that, various stabilizers, the rotational velocities of DC, and different oily media were screened. While polyethylenglycol (PEG 400) and polyvinylpyrrolidone (PVP K17) could not stabilize the aqueous suspension sufficiently and resulted in agglomeration, sodium carboxymethyl cellulose (Na-CMC) resulted in a homogeneous suspension with smallest particle sizes of approx. 370 nm (PDI approx. 0.2) [84]. With the same aim of prolonging drug release, Park et al. developed a subcutaneous applicable nanocrystalline suspension containing montelukast using DC-nanomilling. Polysorbate 80 turned out to be the best stabilizer for the suspension leading to homogeneous suspensions with particles of about 200 nm [85].

To gain a better understanding of the mechanisms of oral bioavailability enhancement, Lynnerup et al. studied the solubility, dissolution behavior, and permeation of DC-made nanoparticles in comparison to the original API powders. However, the equilibrium solubilities of fenofibrate and cinnarizine were not significantly increased by DC-nanomilling, but the dissolution and permeation rates were [86]. In another study, Huang et al. studied the dissolution of nanocrystals, nano-co-crystals, and micro-co-crystals prepared by DC. To achieve the different size levels, indomethacin co-crystals and itraconazole co-crystals were initially prepared by solvent evaporation and then nanomilled with varying DC-milling times. Various stabilizers were tested to gain a stable nano(co-crystal) suspension, identifying Poloxamer 407 (1% *w*/*v*) as the best suitable stabilizer for this preparation [87,88]. The combination of co-crystal formation and DC-based particle size reduction improved both the kinetic solubility and dissolution rate leading, probably, to an improved bioavailability [87].

Recently, Langer at al. used DC to co-nanomill bovine albumin (BSA) and mitotane to obtain albumin-stabilized mitotane nanoparticles (after crosslinking parts of the BSA using glutaraldehyde) for experimental adrenocortical carcinoma treatment, which were still stable after 6 months of storage at 4–8 °C as well as at 20–24 °C [17]. Furthermore, Zhang et al. used DC to nanomill dexamethasone and hydrocortisone, which were loaded subsequently into PLGA microparticles with high EE-values. In in vitro release studies, it was shown that the particle size and the physical state of the entrapped drugs are crucial factors determining the release behavior of the microparticles [89].

Summarizing, the use of DC and horizontally placed vials for nanomilling has some advantages over planetary ball mill approaches as they offer a faster and more gentle process that results in particles comparable to agitator nanomilling [81]. The new technique also allows milling under sterile conditions and can be performed with multiple samples at the same time, which allows efficient screening approaches. After Hagedorn et al. first applied DC to nanomilling in 2017, DC also found increased usage in formulation development in both the pharmaceutical industry and research groups. All applications of DC-nanomilling so far are summarized in Table 2.

## 4. Other DC-Applications in Pharmaceutical Nanotechnology

As well as the applications described above, DC is used in other fields of pharmaceutical nanotechnology. In the field of polymer science, Klein et al. used DC-processing to regulate the length of cylindrical polymer nanostructures, so they become applicable in biomedicine. They were able to demonstrate that fragmentation to a certain length can be better controlled with dual centrifugation than with ultrasonication [93]. Agate et al. used hemp hurds fibers to prepare nanocellulose with DC [94], which can be used as drug delivery systems [95]. Deuringer et al. used DC to develop a simplified method to prepare reconstituted high-density lipoproteins and loaded them with everolimus for the treatment of atherosclerosis and cardiovascular disease [96]. Furthermore, DC was used to encapsulate siRNA in milk-derived extracellular vesicles [97] and to prepare pH-sensitive polyethylene glycol nanoparticles containing allergens for allergen-specific immunotherapy and ovalbumin [98]. Kutza et al. used DC for mixing in order to prepare oil adsorbates with various solid excipients and medium-chained triglycerides as an oily component and compared their characteristics to those resulting from conventional mortar/pestle mixing. The used method affected the adsorbates adsorption properties. Even if the oil was inhomogeneously distributed at the particles with both production methods, DC led to a smoothing of the particle surface, whereas the mortar/pestle blending resulted in an uneven surface and particle destruction [99].

DC is also used outside the production of nanoparticles, e.g., in tissue disruption [100,101], mixing of viscous compounds, and extraction.

## 5. Conclusions

It only took a little trick to also be able to use the well-known dual centrifugation (DC) method for the successful preparation of nanosized pharmaceutical formulations—the horizontal positioning of lengthy sample vials. Based on this small but very important innovation, DC is now a widely accepted technique for in-vial nanomilling and homogenization, which allows the fast, easy, safe, and aseptic preparation of liposomes, emulsions, polymersomes, and solid lipid nanoparticles, as well as nanocrystals.

This review article summarizes the growing innovation in this emerging field, which started over a decade ago with the preparation of liposomes exhibiting unmatched entrapping efficiencies for water-soluble drug compounds. It demonstrates that DC offers several advantages over the established methods for the preparation of nanosized pharmaceutical formulations. In addition to its unmatched simplicity and the ability to process even very small batches, this new technique enables the sterile and safe preparation of pharmaceutical nanoparticles, with the limitation that DC is not suitable for continuous preparation or for upscaling.

The resulting nanoparticles are quite similar to those produced by established manufacturing techniques such as HPH or agitator milling, which significantly accelerates the development of new nanosized pharmaceutical formulations. Another unique feature is the possibility to produce small multilamellar liposomes (SMVs), which has not been achieved by HPH so far. It remains to be seen whether DC will also become established for the preparation of nanosized formulations in a clinical environment for direct patient application (bedside preparation), which would be a tremendous advantage for formulations that have only a limited shelf life.

## Figures and Tables

**Table 2:** Nanocrystals prepared by DC.

Drug	Stabilizers	DC Device	Smallest Median or Mean Particle Size ^1^	Highlights	Reference
Centella asiatica extract	Carbopol 934NF, HPMC E50LV, Kollidon VA64, Kolliphor EL, Kolliphor HS15, Kolliphor RH 40, Lecithin, MC, Na-CMC, PEG 40 stearate, Poloxamer 188, Polysorbate 20, Polysorbate 80, PVP K30, Tyloxapol	ZM 380R	145 nm PDI: 0.28	The optimized nanosuspension formulation for improved skin absorption without occurrence of skin irritations contains centella asiatica extract (10% *w*/*v*), PVP (0.5%), and water (89.5%). HPMC was able to achieve the smallest size after preparation, but PVP exhibited higher stability.	[83]
Cinnarizine	HPMC, SDS	DAC 150	275 nm PDI: 0.29	Particles retained their crystallinity after milling. Nanosizing shows no significant increase in thermodynamic drug solubility but in dissolution rate. A 90 min milling time is identified as the best milling duration for milling speed of 1500 rpm.	[86]
	Poloxamer 188, Polysorbate 20, SDS	DV 1	587 nm	Poloxamer 188 (4%) led to the smallest particles after milling. No change in crystallinity could be observed.	[90]
Curcumin	HPMC, SDS, Polysorbate 80, PVP-VA64	ZM 380R	113 nm	Three of four stabilizer combinations tested led to the same size distribution of nanoparticles.	[68]
Dexamethasone	SDS	ZM 380R	180 nm after freeze-drying	Encapsulation of nanosized drug in PLGA microparticles with high encapsulation efficiencies (>85%) and good release properties.	[89]
Fenofibrate	DOSS, HPMC, Polysorbate 80, PVP-K25, SDS	ZM 380R	127 nm	First use of dual centrifugation for API-nanomilling. No change in the crystal structure during milling. Results of DC-nanomilling are similar to results obtained by agitator mills.	[3,81]
	HPMC, SDS	DAC 150	259 nm PDI: approx. 0.2	Particles retained their crystallinity. No significant increase in thermodynamic drug solubility could be observed but in dissolution rate. The study shows that in vitro dissolution/permeation studies can be employed to better understand oral absorption enhancement of nanocrystal formulations.	[86]
Hydrocortisone	SDS	ZM 380R	160 nm after freeze-drying	The nanosized drugs were encapsulated in PLGA microparticles showing a more continuous release than the micronized drug and a better encapsulation efficiency than with dissolved drug.	[89]
Ibuprofen	HPMC, SDS	ZM 380R	approx. 190 nm	DC-milling of ibuprofen led to smaller particles than milling with a planetary ball mill.	[3,81]
Indomethacin	Poloxamer 188, Polysorbate 20, SDS	DV 1	355 nm	Nanomilling with DC led to highly reproducible results regarding particle size, distribution, and stability. SDS (1%) resulted in the smallest particles sizes after milling. However, nanosuspensions stabilized with poloxamer 188 (4%) showed better short-term stability over 28 days.	[90]
	Poloxamer 407	ZM 380R	163 nm PDI: 0.14 after freeze-drying	The combination of nanocrystals and co-crystals enables a better kinetic solubility and faster dissolution rates compared to the single components.	[87]
Indomethacin -nicotinamide-co-crystals	Poloxamer 407	ZM 380R	280 nm PDI: 0.29 after freeze-drying		[87]
Indomethacin-saccharin-co-crystals	Poloxamer 407	ZM 380R	329 nm PDI: 0.20 after freeze-drying		[87]
Itraconazole	HPC-SL, SDS, Polysorbate 80	ZM 380R	127 nm PDI: 0.18	A combination of three stabilizers at minimal concentrations of 0.9% HPC-SL, 0.14% SDS, and 0.07% polysorbate 80 (all *w*/*w*) was necessary for sufficient stabilization	[82]
	HPC, HPMC E5, Poloxamer 188, Poloxamer 407, Polysorbate 80, PVP K30, SDS, TPGS		223 nm PDI: 0.24 after freeze-drying	PVP and HPMC were not able to form stable itraconazole nanosuspensions	[87,88]
	Poloxamer 407		211 nm PDI: 0.22	Addition of HPMC E5 to the itraconazole nanosuspension did not increase the crystal solubility.	[91]
Itraconazole-fumaric acid-co-crystals	Poloxamer 407	ZM 380R	443 nm PDI: 0.35 after freeze-drying	Itraconazole nano-co-crystals with a size of about 450 nm were successfully prepared for the first time.	[87]
Itraconazole-succinic acid-co-crystals	Poloxamer 407	ZM 380R	455 nm PDI: 0.24 after freeze-drying		[87,92]
	Poloxamer 407	ZM 380R	365 nm PDI: 0.25	Itraconazole-succinic acid-co-crystals can be effectively integrated into an oral solid dosage form using bead layering as a downstream method without negatively affecting the drug dissolution.	[91]
Mitotane	Bovine serum albumin	ZM 380R	359 nm PDI: 0.14	A stable and storable mitotane formulation with a high drug content and good in vitro characteristics was developed.	[17]
Montelukast	Cremophor EL, Cremophor RH40, Na-CMC, PEG 4000, Poloxamer 188, Polysorbate 80, PVP K17, Solutol HS15, Tyloxapol	ZM 380R	190 nm PDI: not indicated	Polysorbate 80 leads to the smallest and most homogeneous particles.	[85]
Naproxen	DOSS, HPMC	ZM 380R	approx. 130 nm	No difference in crystal structure after milling could be observed.	[3,81]
	Polysorbate 80, PVP-K25				[3]
Rotigotine	Kolliphor EL, Kolliphor HS 15, Kolliphor RH 40, Na-CMC, PEG 4000, Poloxamer 188, Polysorbate 80, PVP K17	ZM 380R	375 nm PDI: 0.206	Na-CMC turned out to be the best stabilizer leading to a small particle size with an appropriate homogeneity.	[84]

^1^ Size reported after preparation; size measurements from stability tests are excluded. For measurements with indicated PDI (polydispersity index), the hydrodynamic diameter was determined with DLS (dynamic light scattering); without indication, the mean particle size was determined by laser diffraction. **Abbreviations**: **DOSS**—sodium docusate; **HPC**—hydroxypropyl cellulose; **HPMC**—hydroxypropylmethyl cellulose; **MC**—methylcellulose; **Na-CMC**—carboxymethyl cellulose sodium; **PEG**—polyethylenglycol; **PVP** (-VA64)—poly(1-vinylpyrrolidon (-co-vinylacetat)); **SDS**—sodium dodecyl sulfate; **TPGS**—D-α-tocopheryl polyethylene glycol 1000 succinate.

## Data Availability

Data sharing is not applicable.
